# Evaluation of the Liquid Colony™ Produced by the FAST System for Shortening the Time of Bacterial Identification and Phenotypic Antimicrobial Susceptibility Testing and Detection of Resistance Mechanisms from Positive Blood Cultures

**DOI:** 10.3390/diagnostics13111849

**Published:** 2023-05-25

**Authors:** Chiara Bonaiuto, Ilaria Baccani, Chiara Chilleri, Alberto Antonelli, Tommaso Giani, Gian Maria Rossolini

**Affiliations:** 1 Department of Experimental and Clinical Medicine, University of Florence, 50134 Florence, Italy; chiara.bonaiuto@unifi.it (C.B.); ilaria.baccani@unifi.it (I.B.); chiara.chilleri@unifi.it (C.C.); albertoanton88@gmail.com (A.A.);; 2 Clinical Microbiology and Virology Unit, Careggi University Hospital, 50134 Florence, Italy

**Keywords:** fast microbiology, blood stream infection, rapid CPE detection, rapid AST, rapid ID

## Abstract

Background: the aim of this study was to evaluate the performance of the Liquid Colony™ (LC) generated directly from positive blood cultures (PBCs) by the FAST System (Qvella, Richmond Hill, ON, Canada) for rapid identification (ID) and antimicrobial susceptibility testing (AST) compared with the standard of care (SOC) workflow. Methods: Anonymized PBCs were processed in parallel by the FAST System and FAST PBC Prep cartridge (35 min runtime) and SOC. ID was performed by MALDI-ToF mass spectrometry (Bruker, Billerica, MA, USA). AST was performed by reference broth microdilution (Merlin Diagnostika, Bornheim, Germany). Carbapenemase detection was carried out with the lateral flow immunochromatographic assay (LFIA) RESIST-5 O.O.K.N.V. (Coris, Gembloux, Belgium). Polymicrobial PBCs and samples containing yeast were excluded. Results: 241 PBCs were evaluated. ID results showed 100% genus-level concordance and 97.8% species-level concordance between LC and SOC. The AST results for Gram-negative bacteria showed a categorical agreement (CA) of 99.1% (1578/1593), with minor error (mE), major error (ME), and very major error (VME) rates of 0.6% (10/1593), 0.3% (3/1122), and 0.4% (2/471), respectively. The results from Gram-positive bacteria showed a CA of 99.6% (1655/1662), with mE, ME, and VME rates of 0.3% (5/1662), 0.2% (2/1279), and 0.0% (0/378), respectively. Bias evaluation revealed acceptable results for both Gram-negatives and Gram-positives (−12.4% and −6.5%, respectively). The LC yielded the detection of 14/18 carbapenemase producers by LFIA. In terms of turnaround time, the ID, AST, and carbapenemase detection results were generally obtained one day earlier with the FAST System compared with the SOC workflow. Conclusions: The ID, AST, and carbapenemase detection results generated with the FAST System LC were highly concordant with the conventional workflow. The LC allowed species ID and carbapenemase detection within around 1 h after blood culture positivity and AST results within approximately 24 h, which is a significant reduction in the turnaround time of the PBC workflow.

## 1. Introduction

Sepsis remains a major cause of morbidity and mortality, with important impacts on healthcare systems worldwide [1]. The increased antimicrobial resistance rates present among bacterial pathogens causing sepsis have significantly worsened the healthcare burden by reducing therapeutic options while increasing therapeutic uncertainty [2].

In the diagnostic workup of septic patients, blood culture (BC) is fundamental to identifying the infecting pathogen(s) and determining their antimicrobial susceptibility profiles. This allows for potential adjustment of the initial empiric antimicrobial chemotherapy to the most appropriate regimen in terms of pathogen coverage while minimizing resistance selection by de-escalating broad-spectrum antimicrobial pressure [3].

In the standard BC workflow, the initial detection step in liquid medium is followed by an additional interval of approximately 48 h for species identification (ID) and antimicrobial susceptibility testing (AST). This can result in a substantial delay in the time to administration of definitive antimicrobial therapy [4,5]. This delay can be particularly deleterious when the risk of inappropriate treatment is higher, such as in settings with a high prevalence of multidrug-resistant (MDR) pathogens [6].

In this scenario, novel technologies able to provide faster results from positive BCs (PBCs) are of considerable interest to improve the management of septic patients, optimize antimicrobial therapy, and support antimicrobial stewardship programs [5,6,7].

The aim of this study was to evaluate the FAST System (Qvella, ON, Canada), an automated system for the rapid isolation and concentration of microbial cells directly from PBCs within 35 min. This study differs significantly from previous studies of the FAST System in that AST was performed using a broth microdilution method and a higher percentage of antibiotic-resistant organisms was included. This stronger study design has resulted in the most in-depth evaluation to date of the FAST System, which reduces the time of microbial ID, AST, and the detection of resistance mechanisms for PBCs by approximately one day compared with conventional standard of care (SOC) protocols.

## 2. Materials and Methods

### 2.1. Study Design

In this study, anonymized residual PBCs were processed according to SOC protocols and with the FAST System. Identification and AST results, including carbapenemase resistance, using the FAST System Liquid Colony™ (LC) were compared with overnight subculture results. All BCs had been incubated with the BACTEC ™ FX system (Becton Dickinson, Baltimore, MD, USA) and were processed typically within 2 h and no later than 16 h of positivity. The same bottle was processed with both the SOC and FAST System methods. Polymicrobial samples and samples containing yeast were excluded from the study.

In the SOC workflow, 25 μL of the PBC was subcultured on different solid media for bacterial subculture (Chromid CPSE, Columbia blood agar and Chocolate agar, bioMérieux, Marci L’Etoile, France) and incubated for 18–22 h at 35 ± 1 °C under aerobic and anaerobic atmosphere. Isolated colonies were analyzed by ID, AST, and detection of resistance mechanisms (carbapenemases), as detailed below.

In the FAST System workflow, 2 mL of the residual PBC was transferred into the FAST PBC Prep cartridge. After processing (~35 min for 2 samples), the resulting Liquid Colony™ (LC) was processed for ID, AST, and detection of resistance mechanisms (carbapenemases), as detailed below.

A subculture on the Chromid CPSE medium was carried out from each PBC and bacterial suspension prepared from LC for AST assay and then stored at −80 °C in Brain Heart Infusion broth (Liofilchem, Roseto degli Abruzzi, Italy) supplemented with 20% (*v*/*v*) glycerol for possible repetition of ID, AST, and/or carbapenemase detection in case of discordant results.

### 2.2. Bacterial Identification and Antimicrobial Susceptibility Testing

Bacterial ID was performed by MALDI-ToF mass spectrometry (MS) (Bruker, Billerica, MA, USA). In the SOC protocol, MS was carried out on isolated colonies following the manufacturer’s instructions, using the α-cyano-4-hydroxycinnamic acid (HCCA) matrix. For ID with the FAST System workflow, 2 wells were spotted with 1 µL of LC, followed by addition of formic acid and HCCA matrix after drying.

AST was performed by broth microdilution using commercial plates (ITGP, ITGN, ITHMN, and MICRONAUT-S Anaerobes MIC for nonfastidious Gram-positive, nonfastidious Gram-negative, fastidious, and anaerobic bacteria; Merlin Diagnostika GmBH, Germany). In the SOC protocol, isolated colonies were used to perform AST following the manufacturer’s instructions. For AST with the FAST System workflow, a variable volume of LC (range: 2–64 µL) was added to 2 mL of sterile normal saline to obtain a 0.5 McFarland suspension, and 50 µL (for aerobic species) and 200 µL (for anaerobic species) of the bacterial suspension were added to 11 mL of Mueller–Hinton II broth or Wilkins–Chalgren broth (for anaerobic species previously incubated for 3 h in CO_2_) (Merlin). These suspensions were then used to inoculate (100 µL per well) lyophilized panels. AST results for aerobic and anaerobic species were read after 18 ± 2 and 24–48 h incubation at 35 ± 1 °C in aerobic or anaerobic atmosphere and interpreted according to EUCAST clinical breakpoints v. 13 and v. 11, respectively (https://www.eucast.org/clinical_breakpoints, accessed on 17 May 2023).

### 2.3. Assays for Carbapenemases

The detection of carbapenemases (KPC, VIM, OXA-48, and NDM) was performed by lateral flow immunochromatographic assay (LFIA), carried out using the RESIST-5 O.O.K.N.V system (Coris BioConcept, Gembloux, Belgium) from LC (FAST System workflow) and from isolated colonies (SOC workflow). For the FAST System workflow, 10 µL of LC was added to 12 drops of extraction buffer. The mixture was then vortexed for 15 s, 4 drops were dispensed on each cassette, and results were read after 15 min and interpreted according to the manufacturer’s instructions. Isolated colonies from the SOC workflow were tested according to the manufacturer’s instructions. Carbapenemase gene detection was performed with the BCID2 panel (bioMérieux) on PBC and with real-time PCR targeting *bla*_KPC_, *bla*_NDM,_ *bla*_OXA-48-like_, and *bla*_VIM_ carbapenemase genes [8,9] on isolated colonies.

### 2.4. Comparative Analysis of Results

For ID, concordance between FAST System LC and SOC results was established if the same genus- or species-level identification was achieved, with a score > 1.70, from both methods.

For AST results, categorical agreement (CA), very major error (VME), major error (ME), and minor error (me) rates were evaluated according to the acceptability criteria of ISO 20776-2:2007 [10], while essential agreement (EA) and bias were evaluated according to the updated version of ISO 20776-2:2021 (bias was evaluated only with antimicrobials presenting at least 25 on-scale values) [11].

In case of discrepancies between FAST System LC and SOC results, ID, AST, and carbapenemase detection were repeated with isolated colonies from LC subculture. Unresolved discrepancies were further analyzed by repeated testing from the BC subculture.

## 3. Results

### 3.1. Identification of Bacteria Purified from Positive Blood Cultures with the FAST System

Overall, 241 positive monomicrobial BC specimens, each from a different patient, were processed in parallel by SOC and FAST System protocols. With the SOC protocol, the specimens yielded a total of 119 Gram-positive organisms of 19 different species and 122 Gram-negative organisms of 16 different species. Gram-positives included *Staphylococcus aureus* (N = 22), coagulase-negative staphylococci (CoNS) (N = 71), enterococci (N = 11), streptococci (N = 7), and corynebacteria (N = 4). Gram-negatives included *Escherichia coli* (N = 40), *Klebsiella pneumoniae* (N = 39), other *Enterobacterales* (N = 19), *Pseudomonas aeruginosa* (N = 16), and *Acinetobacter baumannii* (N = 4). A complete list is reported in Supplementary Table S1.

ID from LC obtained with the FAST System was achieved for 226 of the 241 isolates (93.8%). The remaining 15 strains were identified from isolated colonies obtained by both LC and SOC subcultures. Of the 226 samples identified using the LC, 100% exhibited a concordant ID with the SOC at the genus level and 221/226 (97.8%) at the species level. The five species-level discordances involved four samples containing coagulase-negative staphylococci and one sample containing a *Streptococcus oralis* (Supplementary Table S2). All five discordances vs. the SOC protocol were resolved in favor of the SOC when ID was repeated with isolated colonies from LC. A sixth discordance was resolved in favor of the LC result.

### 3.2. Antimicrobial Susceptibility Testing of Bacteria Purified from Positive Blood Cultures with the FAST System

Isolates included in the study exhibited a variety of resistance profiles with the SOC protocol. Methicillin resistance was observed in 5/22 *S. aureus* (22.7%) and 50/71 CoNS (70.4%). Vancomycin resistance was observed in 4/7 *E. faecium* (57.1%). Among *Enterobacterales*, 48/98 (49%) were resistant to extended-spectrum beta-lactamase producers, and 19/98 (19.4%) were also carbapenem-resistant. Among *P. aeruginosa*, 2/16 (12.5%) exhibited a difficult-to-treat resistance (DTR) phenotype. Among *Acinetobacter baumannii*, 4/4 (100%) were carbapenem-resistant. A complete description of susceptibility profiles is reported in Supplementary Tables S3 and S4, respectively.

AST results were obtained for a total of 3255 drug–bug combinations. Compared with the SOC workflow, the AST results using the FAST System LC exhibited CA and EA values of >90% (i.e., acceptable according to ISO 20776-2:2007 and ISO 20776-2:2021) with all tested antibiotics. The AST results for Gram-negative bacteria showed categorical agreement (CA) of 99.1% (1578/1593), with minor error (me), major error (ME), and very major error (VME) rates of 0.6% (10/1593), 0.3% (3/1122), and 0.4% (2/471), respectively. The results from Gram-positive bacteria showed a CA of 99.6% (1655/1662) with mE, ME, and VME rates of 0.3% (5/1662), 0.2% (2/1279), and 0.0% (0/378), respectively. Moreover, mE, ME, and VME values with all molecules fell within the acceptability criteria (≤3%) except for amikacin (AMK) with GNB, which had a VME rate of 5.8% (1/17) (Table 1).

Bias could be estimated only for those antibiotics that were tested with at least 25 isolates with an on-scale MIC. These included amoxicillin/clavulanic acid (AMC), ceftazidime (CAZ), cefepime (CEP), ciprofloxacin (CIP), colistin (COL), ceftolozane/tazobactam (CTA), meropenem (MER), and piperacillin/tazobactam (PIT) for GNB and daptomycin (DPT), trimethoprim/sulfamethoxazole (T/S), teicoplanin (TPL), and vancomycin (VAN) for GPB. With these antibiotics, an overall bias value of −12.4% (range: −1.9/−25.7) and −6.5% (range: −2.4%/−11.2) were observed with GNB and GPB, respectively (Table 1 and Table 2). These values were within the ±30% acceptability rate, although they indicated that using LC showed a tendency to slightly underestimate antimicrobial resistance.

### 3.3. Detection of Carbapenemases in Bacteria Purified from Positive Blood Cultures Using the FAST System LC by LFIA

The detection of carbapenemases by LFIA was performed on 83 samples, which in the SOC workflow yielded 11 KPC producers, 3 NDM producers, 3 KPC/VIM co-producers, 1 VIM producer, and 65 isolates which tested negative for all the searched carbapenemases.

**Figure 1:**
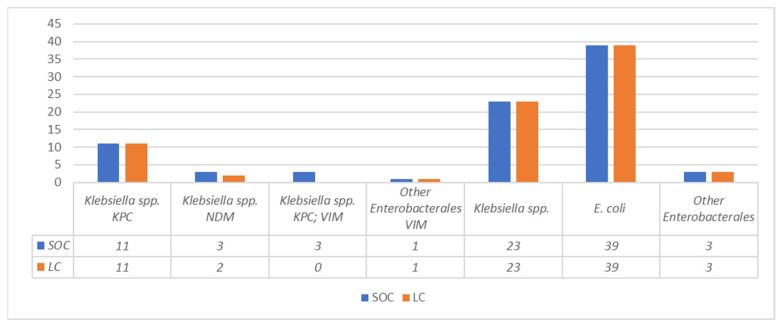
Comparison between LC and SOC CPE detected by LFIA.

When LFIA was performed using the LC obtained by using the FAST System workflow from the same specimens, an overall concordance of 95.2% (79/83) was observed. In particular, with one NDM-producing isolate the carbapenemase was not detected using the LC, and with three KPC + VIM carbapenemase co-producers only the KPC carbapenemase was detected using the LC (Figure 1). Repetition of LFIA with isolated colonies from the LC subcultures resolved all discrepancies.

## 4. Discussion

The FAST System is an automated approach for the rapid isolation and concentration of microbial cells from PBCs [12,13] which decreases turnaround time by approximately 1 day for both ID and AST results. The data presented here reveal an excellent overall concordance of ID and AST results obtained with the LC compared with those obtained following a conventional protocol involving subculturing on solid media and processing of isolated colonies. The results were in overall agreement with those reported by previous studies [12,13,14,15]. Discordant ID results primarily involved PBCs containing coagulase-negative staphylococci, which often contain a mixture of species. Hence, one possible explanation for discordant ID results in such cases is the identification of a dominant population from the LC vs. a minority population from the subculture.

This was the first evaluation of the FAST System LC using reference broth microdilution as an AST method, while previous studies used automated AST systems (e.g., Vitek-2 [12,13] or BD Phoenix [14,15,16] or disk diffusion [15]). Automated systems are often preferred by diagnostic laboratories due to less manual handling and labor. Nevertheless, automated AST systems may exhibit accuracy issues with some antibiotics, especially with MDR pathogens [17,18,19,20], while broth microdilution can also be handled using automated systems. Another original aspect of this study was the inclusion of a higher number of MDR pathogens compared with previous studies [12,13,14,15,16], reflecting the epidemiological setting. Despite the high percentage of MDR pathogens, it is worth noting that no VMEs were observed with Gram-positive bacteria, and only two VMEs were observed with Gram-negative bacteria. Moreover, 1 VME involved *K. pneumoniae* and amikacin: the LC yielded an MIC of 8 (S) while the SOC yielded an MIC of 16 (R). Although this was only a one-dilution MIC difference, which is within the standard error of AST, EUCAST provides no intermediate zone for *Enterobacterales* with amikacin. Both MICs are considered susceptible in the CLSI guidelines (M100).

A further novel aspect of this study was the evaluation of the performance of the rapid LC produced by the FAST System for the detection of carbapenemases with a rapid LFIA method. We showed that the rapid LC obtained from PBCs by the FAST System could be a suitable sample for direct carbapenemase testing by LFIA, at least for detection of some of the most commonly acquired carbapenemases found in *Enterobacterales* (e.g., *bla*_KPC_, *bla*_VIM_, *bla*_NDM_, *bla*_OXA-48_, *bla*_IMP_). Although some cases of VIM and NDM expression were missed by LFIA using the LC, early detection is an important advantage for the management of bloodstream infections involving carbapenemase-producing organisms. In addition, LFIA is a rapid, simple, and inexpensive methodology for the detection of effectors of antibiotic resistance. Immunochromatographic methods, such as the one used here, do not suffer from the limitations typical of genotypic testing, which assess the presence of a gene but not the actual presence of the effector protein nor its amount [21].

Two limitations of this study were its monocentric design and the fact that blood cultures positive for yeast were not evaluated. The rapid identification of pathogens and their resistance genes by molecular syndromic panels is another option to provide rapid results from a PBC [22,23]. However, those systems cover only a limited number of pathogens and resistance mechanisms and, so far, cannot replace the conventional cultural/phenotypic AST approach, which can be significantly shortened using approaches such as the FAST System LC.

The FAST System LC appears to be a useful tool to significantly reduce the turnaround time (TAT) of the conventional PBC workflow to obtain ID and AST (by approximately one day for rapidly growing aerobic bacteria and possibly more for slowly growing fastidious pathogens and anaerobes). It should be noted that such an advantage could be reduced when using a BC workflow that foresees the processing of 4–6 h short subcultures for ID and AST. However, if not operated in a 24 h/7 d regimen, the latter workflow is dependent on the laboratory schedule and can only be performed during the morning shift in laboratories working on an 8–12 h daily basis. In those settings, the FAST System might be considered for processing the PBC samples in the afternoon shift to shorten the time to ID and AST results. A limitation of the FAST System is that polymicrobial infections cannot be processed for ID and AST. A possible alternative approach could be rapid AST by direct inoculation of disk diffusion plates with liquid from PBCs, which has been recommended by EUCAST (https://www.eucast.org/rapid_ast_in_bloodcultures, accessed on 17 May 2023) [24], with three disadvantages: (i) the bacterial concentration of the inoculum is not controlled, (ii) the interpretation is based on inhibition zones which are less accurate than MICs, and (iii) a high percentage of results fall in the intermediate zone, especially with Gram-negative bacteria. The EUCAST RAST approach could potentially benefit from a standardized inoculum obtained with the LC.

Currently, the FAST System has an additional cost of around 60 EUR per test when compared with the conventional workflow. However, the extra cost should be considered in the larger context of the advantages of a reduction in turnaround time of blood culture results in terms of the accuracy of antimicrobial chemotherapy and eventually of improved clinical outcomes, which are likely to result in a shorter length of stay and lower overall cost of care [25,26,27]. In some cases, the use of the FAST system in combination with rapid LFIA might even be considered as a possible substitution of more expensive syndromic panels.

In conclusion, the application of the FAST System Liquid Colony provides accurate ID, AST, and carbapenemase detection results for PBCs while reducing turnaround time by approximately one day, which is especially important in the management of critical care patients with bloodstream infections.

## Figures and Tables

**Table 1:** AST results for GPB.

Gram-Positive Bacteria (N = 119)							
Antibiotics	S (%) *	CA	mE	ME	VME	EA	Bias
		**1655/1662 (99.6%)**	**5/1662 (0.3%)**	**2/1279 (0.2%)**	**0/378 (0%)**	**1645/1662 (99.0%)**	**−6.5%**
AMC **	2/2 (100%)	2/2 (100%)	-	-	-	2/2 (100%)	-
AMP	14/22 (63.6%)	22/22 (100%)	-	-	-	21/22 (95.4%)	-
CFT	22/23 (95.7%)	23/23 (100%)	-	-	-	22/23 (95.6%)	-
CFL	23/23 (100%)	23/23 (100%)	-	-	-	23/23 (100%)	-
CLI	75/106 (70.8%)	105/106 (99.1%)	-	1/75 (1.3%)	-	105/106 (99.1%)	-
DOX	90/94 (95.7%)	93/94 (98.9%)	1/94 (1.1%)	-	-	94/94 (100%)	-
DPT	92/93 (98.9%)	94/94 (100%)	-	-	-	92/93 (98.9%)	−3.5%
ERT **	2/2 (100%)	2/2 (100%)	-	-	-	2/2 (100%)	-
ERY	23/95 (24.2%)	95/95 (100%)	-	-	-	94/95 (98.8%)	-
FUS	57/93 (61.3%)	93/93 (100%)	-	-	-	90/93 (96.7%)	-
GEN	51/93 (54.8%)	93/93 (100%)	-	-	-	92/93 (98.9%)	-
IMP **	2/2 (100%)	2/2 (100%)	-	-	-	2/2 (100%)	-
LEV	43/93 (46.23%)	92/93 (98.9%)	1/93 (1.1%)	-	-	92/93 (98.9%)	-
LIZ	107/109 (98.17%)	109/109 (100%)	-	-	-	109/109 (100%)	-
MER **	2/2 (100%)	2/2 (100%)	-	-	-	2/2 (100%)	-
MOX	52/98 (53.1%)	98/98 (100%)	-	-	-	98/98 (100%)	-
MTR **	2/2 (100%)	2/2 (100%)	-	-	-	2/2 (100%)	-
OXA	38/93 (40.9%)	93/93 (100%)	-	-	-	93/93 (100%)	-
PIT **	2/2 (100%)	2/2 (100%)	-	-	-	2/2 (100%)	-
RAM	81/98 (82.7%)	96/98 (97.9%)	1/98 (1.0%)	1/81 (1.2%)	-	95/98 (96.9%)	-
T/S	86/94 (91.5%)	92/94 (97.9%)	2/94 (2.1%)	-	-	93/94 (98.9%)	−2.4%
TGC	104/104 (100%)	104/104 (100%)	-	-	-	101/104 (97.1%)	-
TPL	106/110 (96.4%)	110/110 (100%)	-	-	-	108/110 (98.2%)	−11.2%
VAN	113/117 (96.6%)	115/115 (100%)	-	-	-	116/117 (99.1%)	−6.1%
TZD	92/93 (98.9%)	93/93 (100%)	-	-	-	93/93 (100%)	-

Percentage values of antibiotic susceptibility (S), category agreement (CA), minor error (me), major error (ME), essential agreement (EA) and bias resulting from Gram-positive isolate assays. AMC: amoxicillin/clavulanate acid; AMP: ampicillin; CFT: ceftobiprole; CFL: ceftaroline; CLI: clindamycin; DOX: doxycycline; DPT: daptomycin; ERT: ertapenem; ERY: erythromycin; FUS: fusidic acid; GEN: gentamycin; IMP: imipenem; LEV: levofloxacin; LIZ: linezolid; MER: meropenem; MOX: moxifloxacin; MTR: metronidazole; OXA: oxacillin; PIT: piperacillin/tazobactam; RAM: rifampicin; T/S: trimethoprim/sulfamethoxazole; TGC: tigecycline; TPL: teicoplanin; TZD: tedizolid; VAN: vancomycin. * Susceptibility rates include isolates susceptible to standard and high-exposure dosing regimens. ** Antibiotics tested only for *Cutibacterium acnes.*

**Table 2:** AST results for GNB.

Gram-Negative Bacteria (N = 122)							
Antibiotics	S (%) *	CA	mE	ME	VME	EA	Bias
		**1578/1593 (99.1%)**	**10/1593 (0.6%)**	**3/1122 (0.3%)**	**2/471 (0.4%)**	**1565/1593 (98.2%)**	**−12.4%**
AMC	47/99 (47.5%)	99/99 (100%)	-	-	-	96/99 (96.9%)	−4%
AMK	101/118 (85.6%)	117/118 (99.1%)	-	-	1/17 (5.9%)	118/118 (100%)	-
CAA	105/114 (92.1%)	114/114 (100%)	-	-	-	106/114 (92.9%)	-
CAZ	63/114 (55.3%)	114/114 (100%)	-	-	-	112/114 (98.2%)	−1.9%
CEP	69/115 (60%)	114/115 (99.1%)	1/115 (0.9%)	-	-	114/115 (99.1%)	−19.5%
CIP	63/119 (52.9%)	118/119 (99.2%)	1/119 (0.8%)	-	-	119/119 (100%)	−4.3%
CLI **	1/1 (100%)	1/1 (100%)	-	-	-	0/1 (0%)	-
COL	108/118 (91.5%)	118/118 (100%)	-	-	-	118/118 (100%)	−25.7%
CRO	49/98 (50%)	97/98 (98.9%)	-	1/49 (2.0%)	-	96/98 (97.9%)	-
CTA	88/114 (77.2%)	113/114 (99.1%)	-	1/88 (1.1%)	-	112/114 (98.2%)	−2.9%
ERT	81/99 (81.8%)	99/99 (100%)	-	-	-	99/99 (100%)	-
GEN	71/102 (69.6%)	102/102 (100%)	-	-	-	102/102 (100%)	-
MER	100/120 (83.3%)	116/120 (96.7%)	3/120 (2.5%)	-	1/20 (5%)	116/119 (97.5%)	−17.9%
MTR **	1/1 (100%)	1/1 (100%)	-	-	-	1/1 (100%)	-
PIT	83/114 (72.8%)	109/114 (95.6%)	5/114 (4.4%)	-	-	110/114 (96.4%)	−13.5%
T/S	51/105 (48.6%)	105/105 (100%)	-	-	-	105/105 (100%)	-
TGC	41/42 (97.6%)	41/42 (97.6%)	-	1/41 (2.4%)	-	40/42 (95.2%)	-

Percentage values of antibiotic susceptibility (S), category agreement (CA), minor error (me), major error (ME), very major error (VME), essential agreement (EA) and bias resulting from Gram-negative isolate assays. AMC: amoxicillin/clavulanate acid; AMK: amikacin; CAA: ceftazidime/avibactam; CAZ: ceftazidime; CEP: cefepime; CIP: ciprofloxacin; CLI: clindamycin; COL: colistin; CRO: ceftriaxone; CTA: ceftolozane/tazobactam; ERT: ertapenem; GEN: gentamycin; MER: meropenem; MTR: metronidazole; PIT: piperacillin/tazobactam; T/S: trimethoprim/sulfamethoxazole; TGC: tigecycline. * Susceptibility rates include isolates susceptible to standard and high-exposure dosing regimens. ** Antibiotics tested only for *Bacteroides fragilis.*

## Data Availability

Not applicable.

## Supplementary Materials

The following supporting information can be downloaded at: https://www.mdpi.com/article/10.3390/diagnostics13111849/s1, Table S1: Isolates included in the study; Table S2: Discrepant identification results between LC and SOC; Table S3: Susceptibility rates of Gram-negative isolates related to Gold Standard assay results; Table S4: Susceptibility rates of Gram-positive isolates related to Gold Standard assay results.

## References

[1] Salomão R., Ferreira B.L., Salomão M.C., Santos S.S., Azevedo L.C.P., Brunialti M.K.C. (2019). Sepsis: Evolving concepts and challenges. Braz. J. Med. Biol. Res..

[2] Iacchini S., Sabbatucci M., Gagliotti C., Rossolini G.M., Moro M.L., Iannazzo S., D’Ancona F., Pezzotti P., Pantosti A. (2019). Bloodstream infections due to carbapenemase-producing *Enterobacteriaceae* in Italy: Results from nationwide surveillance, 2014 to 2017. Eurosurveillance.

[3] Eubank T.A., Long S.W., Perez K.K. (2020). Role of rapid diagnostics in diagnosis and management of patients with sepsis. J. Infect. Dis..

[4] Rhodes A., Evans L.E., Alhazzani W., Levy M.M., Antonelli M., Ferrer R., Kumar A., Sevransky J.E., Sprung C.L., Nunnally M.E. (2017). Surviving sepsis campaign: International guidelines for management of sepsis and septic shock: 2016. Intensive Care Med..

[5] Özenci V., Rossolini G.M. (2019). Rapid microbial identification and antimicrobial susceptibility testing to drive better patient care: An evolving scenario. J. Antimicrob. Chemother..

[6] Leibovici L., Shraga I., Drucker M., Konigsberger H., Samra Z., Pitlik S.D. (1998). The benefit of appropriate empirical antibiotic treatment in patients with bloodstream infection. J. Intern. Med..

[7] Peker N., Couto N., Sinha B., Rossen J.W. (2018). Diagnosis of bloodstream infections from positive blood cultures and directly from blood samples: Recent developments in molecular approaches. Clin. Microbiol. Infect..

[8] Giani T., Antonelli A., Caltagirone M., Mauri C., Nicchi J., Arena F., Nucleo E., Bracco S., Pantosti A., Luzzaro F. (2017). Evolving beta-lactamase epidemiology in *Enterobacteriaceae* from Italian nationwide surveillance, October 2013: KPC-carbapenemase spreading among outpatients. Eurosurveillance.

[9] Coppi M., Antonelli A., Giani T., Spanu T., Liotti F.M., Fontana C., Mirandola W., Gargiulo R., Barozzi A., Mauri C. (2017). Multicenter evaluation of the RAPIDEC^®^ CARBA NP test for rapid screening of carbapenemase-producing *Enterobacteriaceae* and Gram-negative nonfermenters from clinical specimens. Diagn. Microbiol. Infect. Dis..

[10] (2007). Clinical Laboratory Testing and In Vitro Diagnostic Test Systems—Susceptibility Testing of Infectious Agents and Evaluation of Performance of Antimicrobial Susceptibility Test Devices—Part 2: Evaluation of Performance of Antimicrobial Susceptibility Test Devices.

[11] (2021). Clinical Laboratory Testing and In Vitro Diagnostic Test Systems—Susceptibility Testing of Infectious Agents and Evaluation of Performance of Antimicrobial Susceptibility Test Devices—Part 2: Evaluation of Performance of Antimicrobial Susceptibility Test Devices against Reference Broth Micro-Dilution.

[12] Grinberg S., Schubert S., Hochauf-Stange K., Dalpke A.H., Narvaez Encalada M. (2022). Saving time in blood culture diagnostics: A prospective evaluation of the Qvella FAST-PBC Prep application on the Fast System. J. Clin. Microbiol..

[13] Ugaban K., Pak P., She R.C. (2022). Direct MALDI-TOF MS and antimicrobial susceptibility testing of positive blood cultures using the FAST^TM^ System and FAST-PBC Prep Cartridges—Performance evaluation in a clinical microbiology laboratory serving high-risk patients. Microorganisms.

[14] Verroken A., Hajji C., Bressant F., Couvreur J., Anantharajah A., Rodriguez-Villalobos H. (2022). Performance evaluation of the FAST^TM^ System and the FAST-PBC Prep^TM^ cartridges for speeded-up positive blood culture testing. Front. Microbiol..

[15] Novak-Weekley S.M., Khine A.A., Alavie T., Fernandez N., Pandey L., Talebpour A. (2020). 660. Evaluation of Qvella’s FAST-Prep^TM^ Liquid Colony^TM^ for early antimicrobial sensitivity testing of positive blood culture by disk diffusion method. Open Forum Infect. Dis..

[16] Kuo P., LeCrone K., Chiu M., Realegeno S., Pride D.T. (2022). Analysis of the FAST^TM^ system for expedited identification and antimicrobial susceptibility testing of bloodborne pathogens. Diagn. Microbiol. Infect. Dis..

[17] Arena F., Giani T., Vaggelli G., Terenzi G., Pecile P., Rossolini G.M. (2015). Accuracy of different methods for susceptibility testing of gentamicin with KPC carbapenemase-producing *Klebsiella pneumoniae*. Diagn. Microbiol. Infect. Dis..

[18] Antonelli A., Coppi M., Camarlinghi G., Parisio E.M., Nardone M., Riccobono E., Giani T., Mattei R., Rossolini G.M. (2019). Variable performance of different commercial systems for testing carbapenem susceptibility of KPC carbapenemase-producing *Escherichia coli*. Clin. Microbiol. Infect..

[19] Rose D.T., Moskhos A., Wibisono A., Reveles K.R. (2022). Automated susceptibility testing with Vitek 2 compared to MicroScan reduces vancomycin alternative therapy for methicillin-resistant *Staphylococcus aureus* bacteremia. Int. J. Infect. Dis..

[20] Bartoletti M., Antonelli A., Bussini L., Corcione S., Giacobbe D.R., Marconi L., Pascale R., Dettori S., Shbaklo N., Ambretti S. (2022). Clinical consequences of very major errors with semi-automated testing systems for antimicrobial susceptibility of carbapenem-resistant *Enterobacterales*. Clin. Microbiol. Infect..

[21] Salimnia H., Fairfax M.R., Lephart P.R., Schreckenberger P., DesJarlais S.M., Johnson J.K., Robinson G., Carroll K.C., Greer A., Morgan M. (2016). Evaluation of the FilmArray blood culture identification panel: Results of a multicenter controlled trial. J. Clin. Microbiol..

[22] Perez K.K., Olsen R.J., Musick W.L., Cernoch P.L., Davis J.R., Land G.A., Peterson L.E., Musser J.M. (2013). Integrating rapid pathogen identification and antimicrobial stewardship significantly decreases hospital costs. Arch. Pathol. Lab. Med..

[23] Dumkow L.E., Worden L.J., Rao S.N. (2021). Syndromic diagnostic testing: A new way to approach patient care in the treatment of infectious diseases. J. Antimicrob. Chemother..

[24] Åkerlund A., Jonasson E., Matuschek E., Serrander L., Sundqvist M., Kahlmeter G., Dzajic E., Hansen D.S., Agergaard H.N., Pätäri-Sampo A. (2020). EUCAST rapid antimicrobial susceptibility testing (RAST) in blood cultures: Validation in 55 European laboratories. J. Antimicrob. Chemother..

[25] Kumar A., Ellis P., Arabi Y., Roberts D., Light B., Parrillo J.E., Dodek P., Wood G., Kumar A., Simon D. (2009). Initiation of inappropriate antimicrobial therapy results in a fivefold reduction of survival in human septic shock. Chest.

[26] Bassetti M., Kanj S.S., Kiratisin P., Rodrigues C., Van Duin D., Villegas M.V., Yu Y. (2022). Early appropriate diagnostics and treatment of MDR Gram-negative infections. JAC-Antimicrob. Resist..

[27] Mponponsuo K., Leal J., Spackman E., Somayaji R., Gregson D., Rennert-May E. (2022). Mathematical model of the cost-effectiveness of the BioFire FilmArray Blood Culture Identification (BCID) panel molecular rapid diagnostic test compared with conventional methods for identification of *Escherichia coli* bloodstream infections. J. Antimicrob. Chemother..

