# Supplementary material for: Evaluation of the Liquid Colony™ Produced by the FAST System for Shortening the Time of Bacterial Identification and Phenotypic Antimicrobial Susceptibility Testing and Detection of Resistance Mechanisms from Positive Blood Cultures
Source: Diagnostics (Basel). 2023 May 25;13(11):1849. doi: 10.3390/diagnostics13111849 (PMC10253117; doi:10.3390/diagnostics13111849)
Supplement: Supplementary file 1 [file diagnostics-13-01849-s001.zip › diagnostics-2332116-supplementary.pdf]

Supplementary materials:

Supplementary Table S1: Isolates included in the study.

Total collection samples (n=241)	
**Species identification**	**N°**
Acinetobacter baumannii	4
Bacteroides fragilis	1
Citrobacter freundii	2
Citrobacter koseri	1
Corynebacterium amycolatum	1
Corynebacterium minutissimum	1
Corynebacterium striatum	2
Cutibacterium acnes	2
Enterobacter cloacae	2
Enterococcus faecalis	4
Enterococcus faecium	7
Escherichia coli	40
Klebsiella aerogenes	3
Klebsiella oxytoca	2
Klebsiella pneumoniae	39
Listeria monocytogenes	1
Micrococcus luteus	1
Moraxella catarrhalis	1
Morganella morganii	1
Proteus mirabilis	4
Providencia stuartii	1
Pseundomonas aeruginosa	16
Raoultella ornithinolytica	1
Serratia marcescens	2
Staphylococcus aureus	22
Staphylococcus capitis	3
Staphylococcus epidermidis	45
Staphylococcus haemolyticus	7
Staphylococcus hominis	14
Staphylococcus pettenkoferi	2
Stenotrophomonas maltophilia	2
Streptococcus gallolyticus	1
Streptococcus mitis	1
Streptococcus oralis	2
Streptococcus parasanguinis	2
Streptococcus pneumoniae	1

Supplementary Table S2: Discrepant identification results between LC and SOC

	Discrepant identification results				
	**Sample 1**	**Sample 2**	**Sample 3**	**Sample 4**	**Sample 5**
**LC**	*S. capitis*	*S. pneumoniae*	*S. petrasii*	*S. lugdunensis*	*S. epidermidis*
**SOC**	*S. haemolyticus*	*S oralis*	*S. capitis*	*S. haemolyticus*	*S. haemolyticus*

Supplementary Table S3: Susceptibility rates of Gram-negative isolates related to Gold Standard assay results

	**Antibiotics**								
**Strains**		*Klebsiella spp.**	*E. coli*	*Enterobacterales***	*A. baumannii*	*P. aeruginosa*	*S. maltophilia*	*M. catarrhalis*	*B. fragilis*
**N° isolates**		44	40	14	4	16	2	1	1
**% susceptibility**	**AMC**	14/44 (32%)	26/40 (65%)	4/14 (29%)	-	-	-	-	1/1 (100%)
	**AMK**	32/44 (73%)	40/40 (100%)	14/14 (100%)	1/4 (25%)	13/16 (81%)	-	-	-
	**CAA**	38/44 (86%)	40/40 (100%)	13/14 (93%)	-	14/16 (88%)	-	-	-
	**CAZ**	13/44 (30%)	27/40 (68%)	10/14 (71%)	-	-	-	-	-
	**CEP**	13/44 (30%)	28/40 (70%)	12/14 (86%)	-	-	-	1/1 (100%)	-
	**CIP**	17/44 (39%)	22/40 (55%)	10/14 (71%)	-	-	-	1/1 (100%)	-
	**CLI**	-	-	-	-	-	-	-	1/1 (100%)
	**COL**	44/44 (100%)	38/40 (95%)	6/14 (43%)	4/4 (100%)	16/16 (100%)	-	-	-
	**CRO**	13/44 (30%)	28/40 (70%)	9/14 (64%)	-	-	-	-	-
	**CTA**	24/44 (55%)	39/40 (98%)	13/14 (93%)	-	12/16 (75%)	-	-	-
	**ERT**	26/44 (59%)	39/40 (98%)	14/14 (100%)	-	-	-	1/1 (100%)	-
	**GEN**	27/44 (61%)	32/40 (80%)	12/14 (86%)	-	-	-	-	-
	**MER**	27/44 (61%)	40/40 (100%)	14/14 (100%)	-	11/16 (69%)	-	1/1 (100%)	1/1 (100%)
	**MTR**	-	-	-	-	-	-	-	1/1 (100%)
	**PIT**	19/44 (43%)	34/40 (85%)	12/14 (86%)	-	-	-	-	-
	**T/S**	18/44 (41%)	20/40 (50%)	10/14 (71%)	-	-	1/2 (50%)	1/1 (100%)	-
	**TGC**	-	39/40 (98%)	2/2 (100%)	-	-	-	-	-

***including *K. pneumoniae* (n=39), *K. aerogenes* (n=3), *K. oxytoca* (n=2)**

****including *P. mirabilis* (n=4), *E. cloacae* (n=2), *C. koseri* (n=1), *C. freundii* (n=2), *S. marcescens* (n=2), *M. morganii* (n=1), *P. stuartii* (n=1), *R. ornithinolytica* (n=1).**

AMC: amoxicillin/clavulanate acid; AMK: amikacin; CAA: ceftazidime/avibactam; CAZ: ceftazidime; CEP: cefepime; CIP: ciprofloxacin; CLI: clindamycin; COL: colistin;

CRO: ceftriaxone; CTA: ceftolozane/tazobactam; ERT: ertapenem; GEN: gentamycin; MER: meropenem; MTR: metronidazole; PIT: piperacillin/tazobactam;

T/S: trimethoprim/sulfamethoxazole; TGC: tigecycline.

Supplementary Table S4. Susceptibility rates of Gram-positive isolates related to Gold Standard assay results

Strains	Antibiotics	Coagulase-negative staphylococci*	*S. aureus*	*Enterococcus spp.***	*Corynebacterium spp.****	*L. monocytogenes*	*M. luteus*	Viridans streptococci****	*S. pneumoniae*	*C. acnes*
N° isolates		71	22	11	4	1	1	6	1	2
	AMC	-	-	-	-	-	-	-	-	2/2 (100%)
	AMP	-	-	3/11 (27%)	-	1/1 (100%)	1/1 (100%)	6/6 (100%)	-	2/2 (100%)
	CFL	-	21/22 (95%)	-	-	-	-	-	1/1 (100%)	-
	CFT	-	21/22 (95%)	-	-	-	-	-	1/1 (100%)	-
	CLI	48/71 (68%)	20/22 (91%)	-	-	-	1/1 (100%)	4/5 (80%)	-	2/2 (100%)
	DOX	65/71 (92%)	21/22 (95%)	-	-	-	-	-	-	-
	DPT	71/71(100%)	21/22 (95%)	-	-	-	-	-	-	-
	ERT	-	-	-	-	-	-	-	-	2/2 (100%)
	ERY	5/71 (7%)	15/22 (68%)	-	-	1/1 (100%)	-	-	-	-
	FUS	38/71 (54%)	22/22 (100%)	-	-	-	-	-	-	-
	GEN	29/71 (41%)	21/22 (95%)	-	-	-	-	-	-	-
	IMP	-	-	-	-	-	-	-	-	2/2 (100%)
	LEV	-	-	-	-	-	-	-	-	-
% susceptibility	LIZ	69/71 (97%)	22/22 (100%)	11/11 (100%)	4/4 (100%)	-	-	-	1/1 (100%)	-
	MER	-	-	-	-	-	-	-	-	2/2 (100%)
	MOX	29/71 (41%)	20/22 (91%)	-	2/4 (50%)	-	-	-	1/1 (100%)	-
	MTR	-	-	-	-	-	-	-	-	0/2 (0%)
	OXA	21/71 (30%)	17/22 (77%)	-	-	-	-	-	-	-
	PIT	-	-	-	-	-	-	-	-	2/2 (100%)
	RAM	56/71 (79%)	21/22 (95%)	-	2/4 (50%)	-	-	-	1/1 (100%)	-
	T/S	54/71 (76%)	22/22 (100%)	-	-	-	-	-	-	-
	TGC	71/71 (100%)	22/22 (100%)	11/11 (100%)	-	-	-	-	-	-
	TPL	70/71 (99%)	22/22 (100%)	8/11 (73%)	-	-	-	5/5 (100%)	1/1 (100%)	-
	TZD	70/71 (99%)	22/22 (100%)	-	-	-	-	-	-	-
	VAN	100% (71/71)	22/22 (100%)	7/11 (64%)	4/4 (100%)	-	1/1 (100%)	5/5 (100%)	1/1 (100%)	2/2 (100%)

***including *S. epidermidis* (n=46), *S. hominis* (n=14), *S. haemolyticus* (n=4), *S. capitis* (n=3), *S. pettenkoferi* (n=2), *S. lugdunensis* (n=1) and *S. petrasii* (n=1)**

****including *E. faecium* (n=7) and *E. faecalis* (n=4)**

*****including *C. striatum* (n=2), *C. amycolatum* (n=1) and *C. minutissimum* (n=1)**

******including *S. oralis* (n=3), *S. parasanguinis* (n=2) and S. *gallolyticus* (n=1)**

AMC: amoxicillin/clavulanate acid; AMP: ampicillin; CFL: ceftaroline; CFT: ceftobiprole; CLI: clindamycin; DOX: doxycycline; DPT: daptomycin; ERT: ertapenem; ERY: erythromycin; FUS: fusidic acid; GEN: gentamycin; IMP: imipenem; LEV: levofloxacin; LIZ: linezolid; MER:

meropenem; MOX: moxifloxacin; MTR: metronidazole; OXA: oxacillin; PIT: piperacillin/tazobactam; RAM: rifampicin; T/S: tripetromin/sulfametazole; TGC: tigecycline; TPL: teicoplanin; TZD: tedizolid; VAN: vancomycin.
